# A mixture of grass–legume cover crop species may ameliorate water stress in a changing climate

**DOI:** 10.1093/aobpla/plae039

**Published:** 2024-07-25

**Authors:** Nhu Q Truong, Larry M York, Allyssa Decker, and Margaret R Douglas

**Affiliations:** Decarbonization, Unravel Carbon Pte. Ltd., 89 Neil Road #03-03, Singapore 088849, Singapore; Department of Environmental Studies & Environmental Science, 28 N. College St., Dickinson College, Carlisle, PA 17013, USA; Biosciences Division, Oak Ridge National Laboratory, 1 Bethel Valley Rd., Oak Ridge, TN 37830, USA; Department of Environmental Studies & Environmental Science, 28 N. College St., Dickinson College, Carlisle, PA 17013, USA; Department of Environmental Studies & Environmental Science, 28 N. College St., Dickinson College, Carlisle, PA 17013, USA

**Keywords:** cover crops, climate change, diversity, water stress, root morphology, *Secale cereale*, *Trifolium incarnatum*

## Abstract

Climate change models predict increasing precipitation variability in the mid-latitude regions of Earth, generating a need to reduce the negative impacts of these changes on crop production. Despite considerable research on how cover crops support agriculture in a changing climate, understanding is limited of how climate change influences the growth of cover crops. We investigated the early development of two common cover crop species—crimson clover (*Trifolium incarnatum*) and rye (*Secale cereale*)—and hypothesized that growing them in the mixture would ameliorate stress from drought or waterlogging. This hypothesis was tested in a 25-day greenhouse experiment, where the two factors (species number and water stress) were fully crossed in randomized blocks, and plant responses were quantified through survival, growth rate, biomass production and root morphology. Water stress negatively influenced the early growth of these two species in contrasting ways: crimson clover was susceptible to drought while rye performed poorly under waterlogging. Per-plant biomass in rye was always greater in mixture than in monoculture, while per-plant biomass of crimson clover was greater in mixture under drought. Both species grew longer roots in mixture than in monoculture under drought, and total biomass of mixtures did not differ significantly from the more-productive monoculture (rye) in any water condition. In the face of increasingly variable precipitation, growing crimson clover and rye together has potential to ameliorate water stress, a possibility that should be further investigated in field experiments.

## Teaser

Crop production is being impacted significantly by climate change. Cover crops are a promising mitigation and adaptation strategy, but little is known about how their establishment and early growth are affected by climate-related stresses. We conducted a greenhouse experiment to understand responses of rye, crimson clover and their mixture to water stress—an important consequence of climate change in mid-latitude regions. The two species were affected in contrasting ways: waterlogging limited rye's development whereas drought reduced survival and growth of crimson clover. When grown together, the two species demonstrated better performance under drought compared to their monocultures, specifically, longer total root length and comparable or greater total biomass. These results suggest that a rye and crimson clover mixture may increase the likelihood of stable production for farmers, a possibility that merits further investigation under field conditions.

## Introduction

Climate change models project that the mid-latitude region will experience more extreme rain events and droughts by 2035 (IPCC 2013). These precipitation changes will certainly challenge farmers in achieving their production goals, with negative effects expected to increase with precipitation variability (Shortly *et al.* 2015). Longer dry periods can result in dramatic losses to crop production (e.g. for soybean, Leng and Hall 2019), while a higher risk of flooding can delay planting (Shortly *et al.* 2015). Water stress strongly reduces plant growth through several mechanisms. Drought decreases soil moisture, causing a decline in nutrient availability in soil, thereby suppressing plant growth due to a lack of water and nutrients (Studer *et al.* 2017). Meanwhile, waterlogging limits the concentration of oxygen in the soil, slowing root growth or even causing death, which in turn reduces nutrient uptake (Setter and Belford 1990).

Cover cropping is a promising practice that can help farmers mitigate and adapt to climate change while also providing ecosystem services (Clark 2007; Finney and Kaye 2017; Kaye and Quemada 2017). Indeed, cover crops can reduce the risk of soil erosion in extreme rainfall; after being terminated, if they are prepared as mulch, they can preserve water on fields in drought periods (Kaye and Quemada 2017). They have the potential to improve soil health and water quality. A national survey of US farmers reported that improving soil structure and soil health is the leading motivation to adopt cover crops, with 58% (435 of 746 respondents) reporting positive changes in their fields after roughly two years of planting (CTIC 2020). Cover crops often protect the soil, restrict the growth of weeds and conserve or restore nutrients to support the next growing season (Clark 2007). There are several species that are used as cover crops across the USA, among them cereal rye (*Secale cereale*) is the most widely planted (CTIC 2020). Farmers are increasingly interested in cover crop mixes; 46% (406 of 893 respondents) planted mixtures in 2019 and decided to continue using them in 2020 (CTIC 2020). Moreover, the choice of how many species to blend varied widely from two to eight species, but mixing two species was the most common recipe (CTIC 2020). Grass–legume mixtures are particularly popular because they combine the high biomass production of grasses with the ability of legumes to fix nitrogen for the following crop (Clark 2007; Reiss and Drinkwater 2020).

Plant biodiversity is expected to improve biomass productivity, which arises through two ecological mechanisms: (i) sampling effect and (ii) niche complementarity (Loreau 2000). The sampling effect suggests that the better performance of mixtures results from a higher chance of having productive species in a diverse community compared to a monoculture population (Heijden *et al.* 1999). On the other hand, niche complementarity explains that a mixture’s better performance is due to a more effective use of resources (Flombaum *et al.* 2014). Underlying this increased resource use efficiency are two mechanisms: (i) resource partitioning in which species with different traits use resources differently, and (ii) facilitation in which one species alters the environment in a way that supports others (Flombaum *et al.* 2014). Finally, the stress-gradient hypothesis suggests that positive interactions among plant species are likely to increase with increasing environmental stress (He *et al.* 2013).

In cover crops, empirical support has been mixed and context-dependent for the diversity-productivity relationship and its predicted benefits for agroecosystem services (Smith *et al.* 2014; Finney *et al.* 2016; Florence and McGuire 2020; Reiss and Drinkwater 2020; Blanco-Conqui 2023). A systematic review found little evidence that cover crop mixtures outperform monocultures: in 88% of studies, the best-performing mixture and the best-performing monoculture yielded comparable results across several metrics including biomass production, weed suppression, and N retention (Florence and McGuire 2020). Increased plant diversity also did not, in most cases, improve soil physical health compared to monocultures (Blanco-Canqui 2023). That said, benefits have been observed in specific species mixtures under specific conditions. Previous research on grass–legume mixtures suggests that they can increase productivity in some contexts (Reiss and Drinkwater 2020), especially under poor soil fertility (Reiss and Drinkwater 2022). Drought has been shown to strengthen the diversity-productivity relationship in a natural plant community (Mulder *et al.* 2001). To the best of our knowledge, this possibility has not been explicitly tested in cover crops in general or in grass–legume mixtures in particular.

Belowground interaction in cover crop mixtures is under-investigated, as aboveground productivity has long been the main metric used to evaluate cover crops (Blanco-Canqui 2023). Root morphology encompasses an important set of traits underlying plant response to water stress, and potentially mediating the productivity–diversity relationship. Howell *et al.* (2019) found that plants exhibiting resistance to drought and waterlogging have a longer root length and a larger root diameter at a specific soil depth. Indeed, as these root traits increase, the root system can better acquire moisture and nutrients, allowing the plant to tolerate drought and waterlogging (Howell *et al.* 2019). Work in the ‘three sisters’ polyculture of maize, bean and squash showed that the three species complement one another through vertical niche segregation and better utilization of resources available throughout the soil profile (Zhang *et al.* 2014). The mechanism for this complementarity was further substantiated using simulation modelling that showed similar results that could only be the result of reduced competition and more efficient soil use (Postma and Lynch 2012). When mixing species, Mariotti *et al.* (2009) observed belowground competition between cereals and legumes, specifically a decrease in root:shoot ratio of both species, which further influenced above-ground biomass—cereals increased in shoot growth, and legumes reduced in shoot dry weight. However, with facilitation effects, mixtures overcame competition, producing a higher relative total yield than monocultures (Mariotti *et al.* 2009).

While the practice of growing cover crops to address climate change has been extensively investigated (Kaye and Quemada 2017), the influence of climate change on cover crops and their effective management under changing conditions remain unclear. Ensuring successful establishment is particularly important: this is one of the largest obstacles to cover crop adoption in the northern USA, given the short establishment window following the harvest of main-season crops (CTIC 2020). In the Northeast, total precipitation historically increased in warm seasons (June–October), which include the establishment period of cover crops (i.e. August–September) (Frei *et al.* 2015; Huang *et al.* 2017). However, climate projections also predict that droughts will occur more often in the autumn by mid-century (Shortly *et al.* 2015). Given the uncertainty of climate change, we investigated the impacts of water stress on the early development of cover crop species in both directions: water scarcity and excess water. Considering ecological theory predicts improved performance under environmental stress with plant diversity, we further explored if growing in a mixture could ameliorate the influence of water stress on cover crop species in their early development. This research focuses on two common cover crop species in Pennsylvania: crimson clover (*Trifolium incarnatum*) and cereal rye (*Secale cereale*) (CTIC 2020). We hypothesized that the early growth of these cover crop species would be affected by water stress, but the impacts would be reduced when the two species were grown together. We tested this hypothesis in a greenhouse experiment focusing on crop establishment. The greenhouse setting allowed us to carefully control water conditions and collect detailed data elucidating mechanisms underlying observed responses, including survival, growth rate, biomass and root morphology. Results are interpreted for evidence of the sampling effect and niche complementarity.

## Materials and Methods

### Greenhouse setting

**Figure 1.**
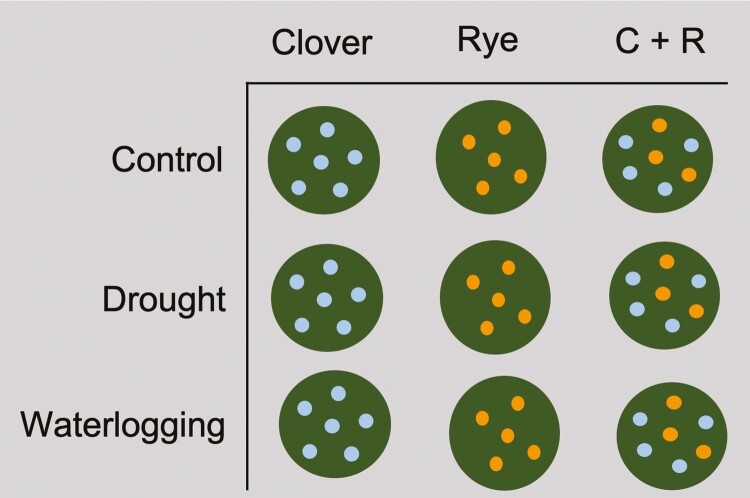
One replicate block of the experiment’s randomized block design.

To better understand the effects of water stress and species number on cover crops’ early development, we conducted an experiment in the Dr Inge P. Stafford Greenhouse of Dickinson College (Carlisle, PA, USA) starting in June 2021. The experimental design was a randomized block design with a total of four blocks; in each block, two factors—water stress and species number—were fully crossed (Fig. 1). Two investigated cover crop species, crimson clover and rye, were cultivated from seeds in polyvinyl chloride (PVC) pots (18 cm in height and 16 cm in diameter) filled with coarse Turface^®^ MVP^®^ clay (Profile Products LLC., Buffalo Grove, IL, USA). This growing medium was mixed with 45 g of organic fertilizer 3-4-4 (Garden-tone, The Espoma Company, Millville, NJ, USA). Turface^®^ had a bulk density of 577 ± 32 kg m^-3^ and was used to facilitate the examination of root morphology (Goron *et al.* 2015).

The species number treatments comprised a mixture of crimson clover and rye, and their corresponding monocultures. Seeding rates followed field recommendations by Clark (2007): (i) monoculture crimson clover (six plants per pot; 15–20 lb./A), (ii) monoculture rye (five plants per pot; 60–120 lb./A), and (iii) biculture rye and crimson clover (three rye plants and four crimson clover plants per pot). Rye was seeded directly while crimson clover was inoculated beforehand (N-Dure^TM^ Alfalfa/True Clover, Verdesian, Cary, NC, USA). To ensure successful germination, we sowed double the target number of seeds in every pot. On the 7th day after seeding, seedlings were thinned and transplanted among pots in order for all pots to obtain the desired number. Compared to the targeted seedling numbers, transplants accounted for 7% of monoculture crimson clover, 3% of biculture rye and 29% of biculture crimson clover. Transplanting was carried out in the early evening when the light intensity and air temperature were low; we also covered the greenhouse roof for 24 hr after transplanting to reduce heat stress on transplanted seedlings.

The temperature of the greenhouse was set to 27 °C/16 °C for day/night to represent the normal maximum and minimum conditions in Carlisle, PA, USA during the establishment period of cover crops—between August and September (1991–2020 normals for August and September were 29 °C/16 °C and 25 °C/12 °C, respectively, NOAA 2022). The Turface^®^ and ambient temperatures were logged daily using HOBO data loggers (Appendix A, Fig. 7). The sensors measuring Turface^®^ temperature were situated 4.5 cm below the surface, while sensors logging ambient temperature were situated 10 cm above the surface. The pots where sensors were placed received the same water level as the controls.

**Figure 2.**
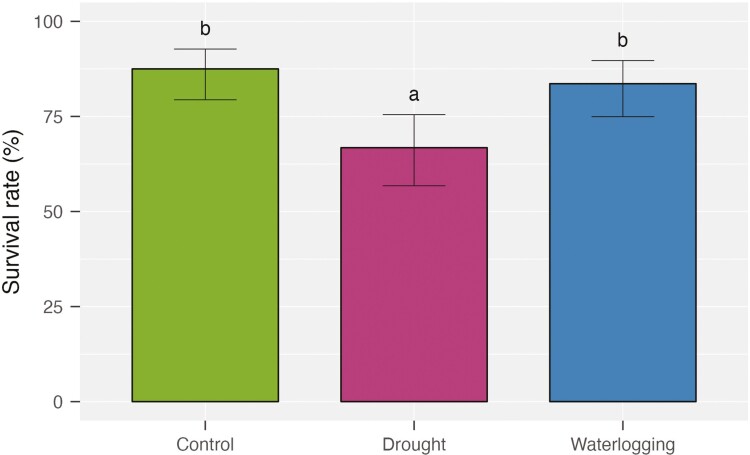
Survival rate (mean ± 95% confidence interval) of crimson clover seedlings under three water treatments at the end of a greenhouse experiment testing the influence of water stress on early growth of cover crop monocultures and bicultures (*n* = 8 pots per water treatment, survival averaged over levels of species number).

**Figure 3.**
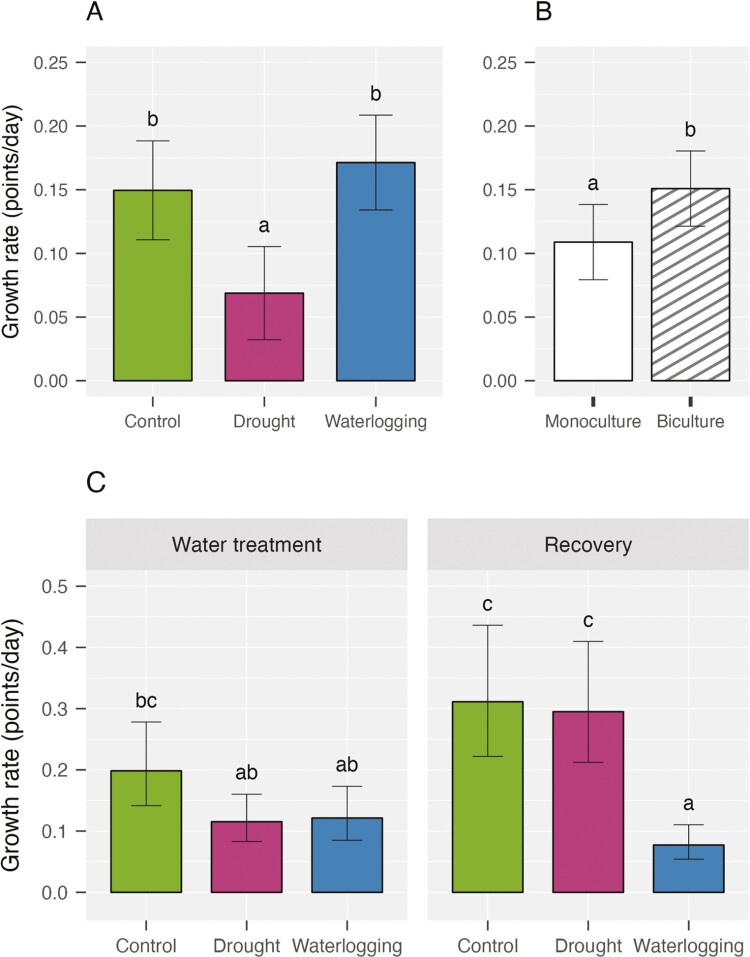
Growth rate (mean ± 95% confidence interval) of (A) crimson clover under water stress (*n* = 8 pots per water treatment), (B) crimson clover by species number (*n* = 12 pots per species number), and (C) rye (mean ± 95% confidence interval) under water stress during the water treatment and recovery phases (*n* = 8 pots per water treatment). (A) and (B) are shown combined across water treatment and recovery phases because there was no significant effect of phase in this species (see Table 1).

**Figure 4.**
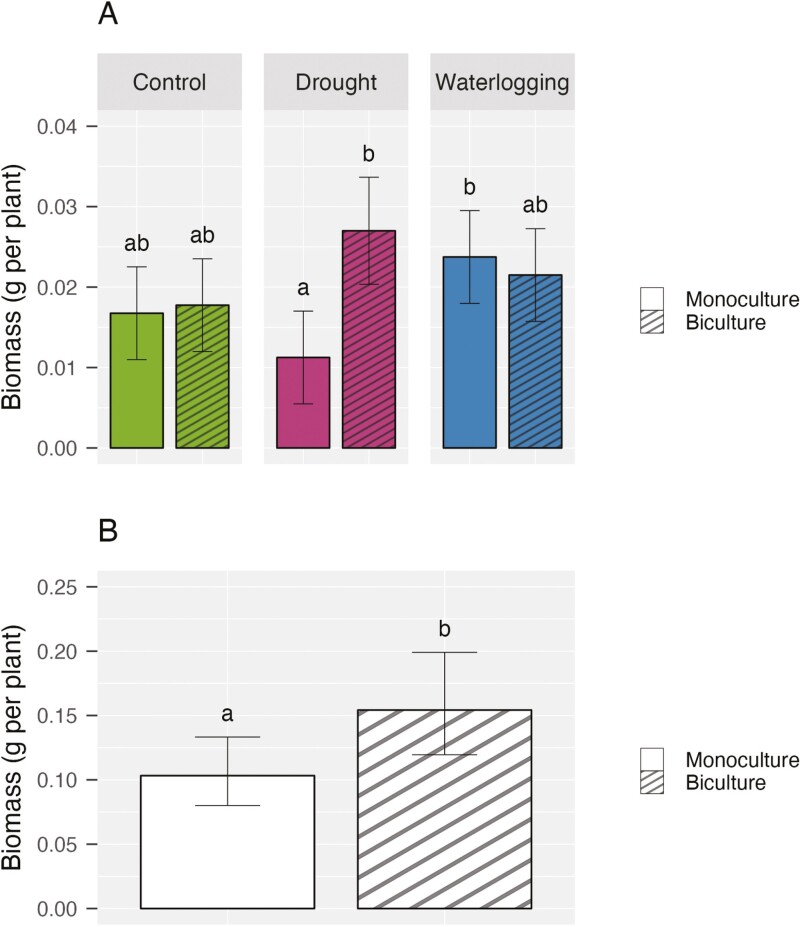
(A) Total biomass of crimson clover (mean ± 95% confidence interval) under water stress by species number (*n* = 4 pots per water-species number combination). (B) Total biomass of rye (mean ± 95% confidence interval) by species number (*n* = 12 pots per species number).

**Figure 5.**
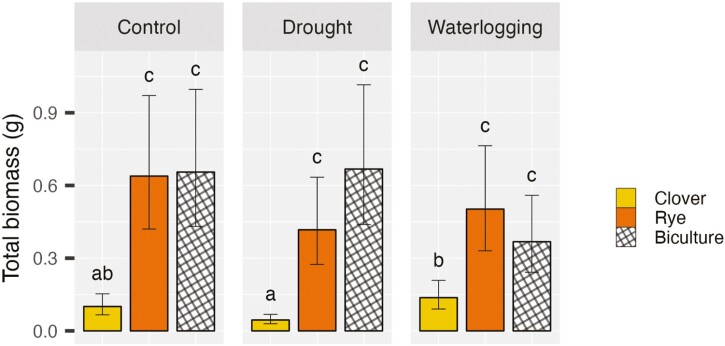
Total biomass per pot (mean ± 95% confidence interval) by water stress and species number treatment (*n* = 4 pots per water-species number combination).

**Figure 6.**
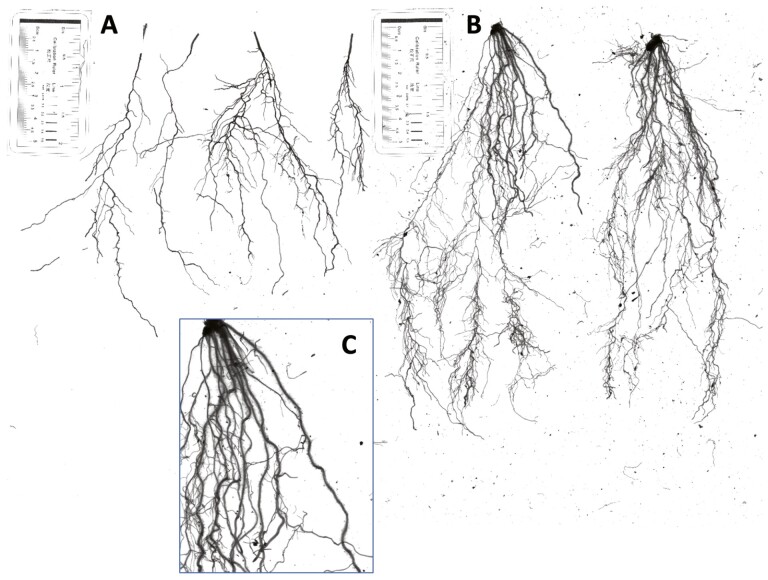
(A and B) Intact root systems of drought biculture crimson clover and rye, respectively. A ruler is included in the intact root images (i.e. the first scan) as a standard scale to relatively compare the roots, but it was not included in the separated root images (i.e. the second scan) which were later formally analysed in the RhizoVision Explorer. (C) Root hairs along the fine roots in rye.

### Water stress imposition

Water stress imposed in the study consisted of three water levels: (i) control with 100% of field capacity, (ii) drought of no irrigation, and (iii) waterlogging where the water level was 2–5 mm above the surface to partly submerge the seedlings (Kaur *et al.* 2020; Yang *et al.* 2020). The field capacity of Turface^®^ was measured prior to the start of this experiment following Imakumbili (2019). We watered three replicates containing the same amount of air-dried Turface^®^ until the pots started leaking, then covered the pots with plastic bags, allowing only downward drainage. After three days, we collected 100 g of Turface^®^ in the centre of each pot. By comparing the mass of Turface^®^ when saturated and after oven-dried, we calculated the medium’s field capacity to be 53.13% volumetric soil moisture, which was equivalent to 930 g of water for 1750 g of Turface^®^—the mass of Turface^®^ in each pot in the experiment—to reach 100% field capacity.

Pots in all water treatments were maintained at 100% field capacity in the first two weeks. When the water treatments were initiated on the 13th day after seeding, drought pots were withheld water, and waterlogging pots were lined with a plastic bag to cover drainage holes and left flooded at about 5 mm above the surface. Following water stress experiments conducted by Kaur *et al.* (2020), Garcia *et al.* (2020), and Yang *et al.* (2020), the water treatment took place for one week, followed by a recovery phase. In detail, on the 20th day after seeding, the water levels of all pots were brought back to 100% field capacity, and the plants were allowed to recover for 5 days before being harvested. Throughout the experiment, we maintained the assigned water levels by weighing pots and irrigating daily (Ogbaga *et al.* 2020). Additionally, to avoid the influence of pot positions on the examined effects, pots were rotated daily within each block in the greenhouse (Bezuidenhout *et al.* 2012).

### Growth score and survival rate

To capture changes in seedlings under the water treatments and in the recovery from stress, we recorded their growth daily. For clover, early growth is generally categorized into three stages of emergence, leaf production, and branching (Mills 2017). Adapting these three main vegetative stages, we quantified growth as the emergence of a new set of leaves. Thus, we set the growth stages of clover as emergence of cotyledons, first true leaf, first trifoliate leaf, second trifoliate leaf, etc., and each stage was equivalent to one point. For rye, according to the ‘leaf collar method’, rye seedlings gained one point for each visible leaf collar (Abendroth *et al.* 2011). Growth points reflected the total number and/or types of leaves produced regardless of wilting.

### Root trait analysis

On the 25th and 26th day after seeding, the experiment was terminated, and all plants were harvested. We carefully removed plants from the Turface^®^ MVP^®^ and washed them with tap water. Their shoots and roots were separated and stored by replicate and species. The shoots were oven-dried at 60 °C for 48 hr and then weighed. The roots were placed in a plastic bag and stored at 4 °C while we processed the remaining roots. When all samples were completely processed, each group of roots were floated in 400 mL of water on an 8 × 10 in transparent tray and scanned with EPSON Pro V850 flatbed scanner at a resolution of 800 dpi (Sofi *et al.* 2018). The roots were divided into smaller parts and spread for scanning. Scanned roots were then oven-dried at 60 °C for 48 hr and weighed with their dry shoots to obtain a total dry biomass. The scanned images were analysed with RhizoVision Explorer v2.0.3 under the ‘broken root’ mode (Seethepalli and York 2020), based on the methods described by Seethepalli *et al.* (2021). In the settings, we optimized the analysis of root systems. To capture very fine roots, we created five root diameter ranges distributed from 0 to 2 mm, and a last range from 2 mm and above. For crimson clover, we excluded root hairs by setting the image threshold to 200 and root pruning to 5. Since rye roots were highly dense and clumped, we further reduced the threshold to 196 and increased the pruning to 8.

### Statistical analysis

All indicators of growth were analysed by species with statistical tests in R 4.1.0 (R Core Team 2021). The general model that we employed was:


$$\begin{array}{l} Response∼water\;stress\;x\;species\;number \end{array}$$

Effects were considered statistically significant at *P* < 0.05. Where significant effects occurred, we conducted post-hoc pairwise comparisons of means using Tukey’s ‘Honest Significant Difference’, which includes a correction for multiple comparisons (Quinn and Keough 2002; Aho 2013). Data was first fitted with two-way ANOVA. When the homogeneity assumption was violated despite implementing general transformations such as log and square root, we applied generalized linear models (GLM), with the distribution family chosen based on data type and patterns observed in residual versus fitted plots (Zuur *et al.* 2007).

Survival rate was the percentage of target seeds in a particular pot that were alive at the end of the experiment. Since it was discrete data, we applied generalized linear models (GLM) with a quasi-binomial distribution (Zuur *et al.* 2007).

The analysis of growth rate required first estimating this value for each replicate using linear regression. Growth for each replicate-species combination was divided into two phases: water treatment (day 13th–19th after seeding) and recovery (days 19th–26th after seeding). Linear regressions for each subset of the data were fit to the form:


$$\begin{array}{l} Growth\;points∼date \end{array}$$

Slopes from the models (corresponding to shifts in points per day, *n* = 48 per species) became the response variable for a two-way ANOVA of the form:


$$\begin{array}{l} Growth\;rate∼water\;stress\;x\;species\;number\;x\;phase\;+\;starting\;points \end{array}$$

This model also included a random effect of pot ID to account for repeated measurements over time, which was accommodated by fitting the model using the function ‘lme’ (Pinheiro and Bates 2023). The growth rates of rye were log-transformed to improve homoscedasticity.

Total dry biomass per plant for each species was determined by adding plant components (i.e. shoot and root) of the species in a particular pot, then dividing by its number of seedlings. To improve homoscedasticity, we applied generalized linear models (GLM), and family Gamma (Zuur *et al.* 2007). In addition to examining each species separately, to compare total yield we analysed total dry biomass per pot under a similar model. Species composition was categorized differently compared to previous tests; specifically, it was divided into three treatments: monoculture crimson clover, monoculture rye and biculture.

To understand the influence of water and species number on the root morphology of each species, we conducted several interrelated analyses starting with a principal component analysis (PCA). First, root traits generated from RhizoVision Explorer were examined for correlations using the ‘corrplot’ package in R. We selected a subset of strongly correlated traits to simplify the variables in further analyses and included other relevant growth indicators to understand their relationships to root morphology. The analysed parameters were root:shoot mass ratio, average total biomass (grams per plant), number of root tips per plant, total root length (millimeters per plant), branching frequency, average diameter, maximum diameter, root length of five diameter ranges (millimeters per plant), root length ratio of very fine roots to the entire root and root length ratio of very fine to fine roots. For each of the first two principal components, we identified the most influential traits as those accounting for the maximum variability. We visualized the results by mapping each replicate on a plot defined by the two first principal components, and computed ellipses to represent the 95% confidence intervals for each treatment combination of water stress and species number. Following the PCA, we performed two-way ANOVAs on the most influential traits following the same logic as the previous analyses.

## Results

### Survival rate

The survival rate of crimson clover varied significantly under water stress (Table 1). On average, about 65% of seedlings survived under drought (Fig. 2), compared to more than 80% of seedlings under control and waterlogging (Fig. 2).

**Table 1.** Statistical results from analyses of per-plant responses of cover crops to water stress and species number in a fully crossed greenhouse experiment (*n* = four pots per species number-water treatment combination). Growth rate was analysed in two phases: during water stress (7 days) and recovery (7 days).

Indicator	Treatment effect	Crimson clover				Rye			
		dfNum	dfDen	*F*	*P* ^*^	dfNum	dfDen	*F*	*P* ^*^
Survival rate (GLM, quasi-binomial)^†^^,^^‡^	Water	2	18	7.27	**0.010**	2	18	1.18	0.330
	Species number	1	18	0.82	0.378	1	18	1.19	0.289
	Water × species number	2	18	0.71	0.506	2	18	1.07	0.365
Growth rate (two-way ANOVA)^†^^,^^‡^	Water	2	36	9.63	**0.002**	2	36	4.01	**0.037**
	Species number	1	36	4.69	**0.045**	1	36	5.59	**0.030**
	Phase	1	36	2.79	0.111	1	36	9.71	**0.006**
	Start stage	1	36	4.17	**0.057**	1	36	17.05	**0.001**
	Water × species number	2	36	0.52	0.606	2	36	2.24	0.137
	Water × phase	2	36	1.65	0.22	2	36	16.44	**<0.001**
	Species number × phase	1	36	0.20	0.663	1	36	0.013	0.911
	Water × species number × phase	2	36	0.40	0.674	2	36	0.41	0.670
Biomass (two-way ANOVA^†^/GLM, gamma^‡^)	Water	2	17	2.24	0.137	2	18	2.24	0.135
	Species number	1	17	3.52	0.078	1	18	5.26	**0.034**
	Water × Species number	2	17	5.56	**0.014**	2	18	2.14	0.146
Root length (GLM, gamma)^†^^,^^‡^	Water	2	18	6.42	**0.008**	2	18	2.08	0.154
	Species number	1	18	2.59	0.125	1	18	6.00	**0.025**
	Water × species number	2	18	6.69	**0.007**	2	18	4.38	**0.028**
Average/maximum diameter (ANOVA)^†^^,^^‡^	Water	2	18	0.17	0.843	2	18	0.32	0.733
	Species number	1	18	0.04	0.840	1	18	1.07	0.315
	Water × species number	2	18	0.03	0.968	2	18	0.20	0.823

^*^Bold values are significant at α = 0.05.

^†^Statistical tests performed on crimson clover’s data.

^‡^Statistical tests performed on rye’s data.

Contrary to clover, rye maintained the original number of seedlings under all treatments by the end of the experiment, except for one replicate under drought where only 60% of seedings survived. There were no significant differences among treatments (Table 1).

### Growth rate

From the implementation of water stress to the end of recovery, the growth rate of crimson clover was significantly affected by both water stress and species number, but not their interaction (Table 1). The seedlings took significantly longer to grow a new set of leaves under drought. Indeed, their average growth rate was suppressed by 46% and 60% compared to the control and waterlogging, respectively (Fig. 3A). Moreover, growing in a mixture increased clover’s growth rate on average by roughly 40% compared to clover grown in monocultures (Fig. 3B).

Contrasting with crimson clover which was more susceptible to drought, rye was significantly impacted by waterlogging. A significant interaction between water stress and phase in its growth rate was exhibited (Table 1). At the conclusion of the water treatment, growth rate under drought more than doubled, while under waterlogging the growth rate did not change significantly, with a trend toward decline (log-transformed) (Fig. 3C). Furthermore, we found a significant main effects of species number (Table 1). The species’ growth rate in the mixture was 31% higher than that in monoculture.

### Total biomass per plant

Crimson clover’s per-plant biomass was significantly influenced by an interaction between water stress and species number. Specifically, under drought, the biculture clover accumulated 2.4 times more biomass per plant than its corresponding monoculture (Fig. 4A). However, the biculture clover produced as much biomass (0.022 g per plant) as its monoculture (0.024 g per plant) under waterlogging (Fig. 4A). For rye, the mixture increased per-plant biomass by 50% compared to the monoculture (Fig. 4B). This influence was statistically significant regardless of the water treatment. In addition, we noticed a non-significant trend regarding water stress: rye seedlings under waterlogging had a per-plant biomass that was 38% smaller than the control on average.

### Total biomass per pot

Total biomass per pot was statistically affected by an interaction between water stress and species composition (Table 2). The yield of monoculture crimson clover was consistently smaller than both monoculture rye and the rye-clover biculture under all three water levels. Indeed, the total biomass of monoculture crimson clover was on average 6.4 times and 8.0 times lower than that of monoculture rye and biculture, respectively (Fig. 5). Within monoculture crimson clover, pots experiencing waterlogging yielded 3.1 times more than pots under drought (Fig. 5). It was also observed that monoculture rye pots had a 37% greater biomass than biculture pots under waterlogging while producing 38% less under drought (Fig. 5). However, Tukey post-hoc tests found no statistical differences between these treatments (Fig. 5).

**Table 2.** Responses of total biomass per pot (rye and crimson clover combined) in a greenhouse experiment with fully crossed water stress and species number (*n* = 4 pots per species number combination).

Indicator	Treatment effect	dfNum	dfDen	*F*	*P* ^*^
Total biomass (GLM, gamma)	Water	2	27	2.16	0.135
	Species number	2	27	56.64	**<0.001**
	Water × species number	4	27	4.36	**<0.001**

^*^Bold values are significant at *α* = 0.05.

### Root analysis

Regardless of water stress, crimson clover developed shorter roots relative to rye (Fig. 6A). Moreover, crimson clover’s roots were less dense and had less extensive root hairs (Fig. 6B and C). The scan images were then imported to the RhizoVision Explorer program to generate root traits which were then analysed in PCA.

**Figure 7.**
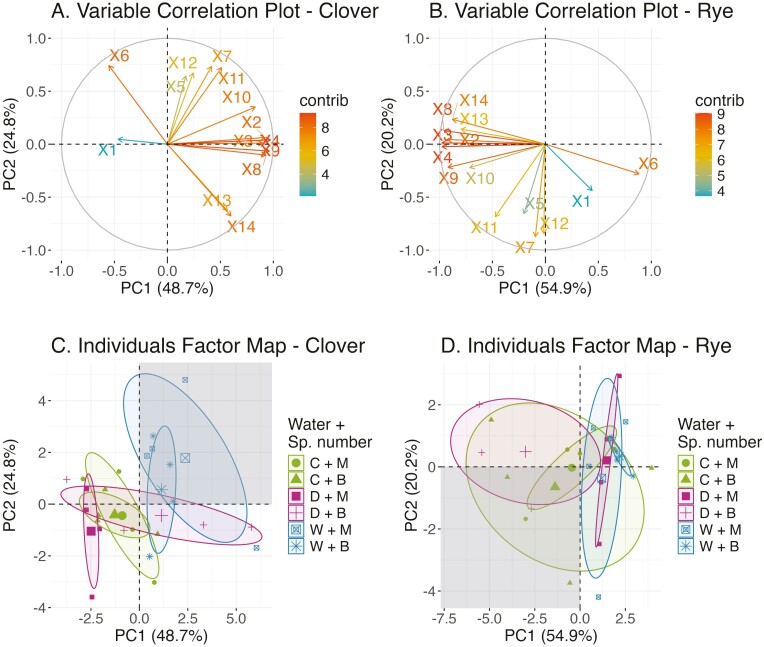
(A and B) Variable correlation plot for crimson clover and rye, respectively. X1: root:shoot mass ratio, X2: total biomass (gram per plant), X3: number of root tips, X4: total root length, X5: branching frequency, X6: average diameter, X7: maximum diameter, X8: root length of diameter range 0–0.25 mm, X9: root length of diameter range 0.25–0.5 mm, X10: root length of diameter range 0.5–1 mm, X11: root length of diameter range 1–2 mm, X12: root length of diameter that is greater than 2 mm, X13: root length ratio of very fine to the entire root, X14: root length ratio of very fine to fine root. (C and D) Individuals factor map for crimson clover and rye, respectively. Shaded quartiles are considered favourable characterizing increases in total root length and diameter. Each ellipse represents a combined treatment of water stress and species number (*n* = 4 pots per species number combination). C + M: control + monoculture; C + B: control + biculture; D + M: drought + monoculture; D + B: drought + biculture; W + M: waterlogging + monoculture; W + B: waterlogging + biculture.

The first two principal components (PC1 and PC2) accounted for 73.5% and 75.1% of the variability in crimson clover and rye root data, respectively (Fig. 7). Among examined traits, the factor loading values showed that total root length and root diameter explained the most variance of the first two principal components, respectively, in both species (Fig. 7A and B). In crimson clover the second principal component was most associated with average root diameter, while in rye it was associated with maximum root diameter.

Our results agreed with previous studies where researchers identified longer root lengths and larger root diameters as key adaptive responses to water stress (Sofi *et al.* 2018; Howell *et al.* 2019). We, therefore, defined for each PCA plot the quartile where seedlings had a longer total root length and a larger diameter as the most favourable quartile and the quartile observing the reverse as the least favourable quartile (Fig. 7C and D). We further determined that a quartile becomes less favourable as the second and first principal components decrease subsequently (Fig. 7C and D).

For clover, the waterlogging monoculture and biculture resided in the favourable area, indicating that they had established robust root systems (Fig. 7C). This species’ drought biculture fell in the second most favourable quartile (+PC1, −PC2). Meanwhile, its drought monoculture occupied the least favourable quartile. For rye, robust roots were mainly shown in the control monoculture and biculture, followed by the drought biculture which resided mainly in the second most favourable quartile (−PC1,+PC2) (Fig. 7D). On the other hand, the waterlogging of both species number treatments and the drought monoculture ellipses occupied the two least favourable areas.

**Figure 8.**
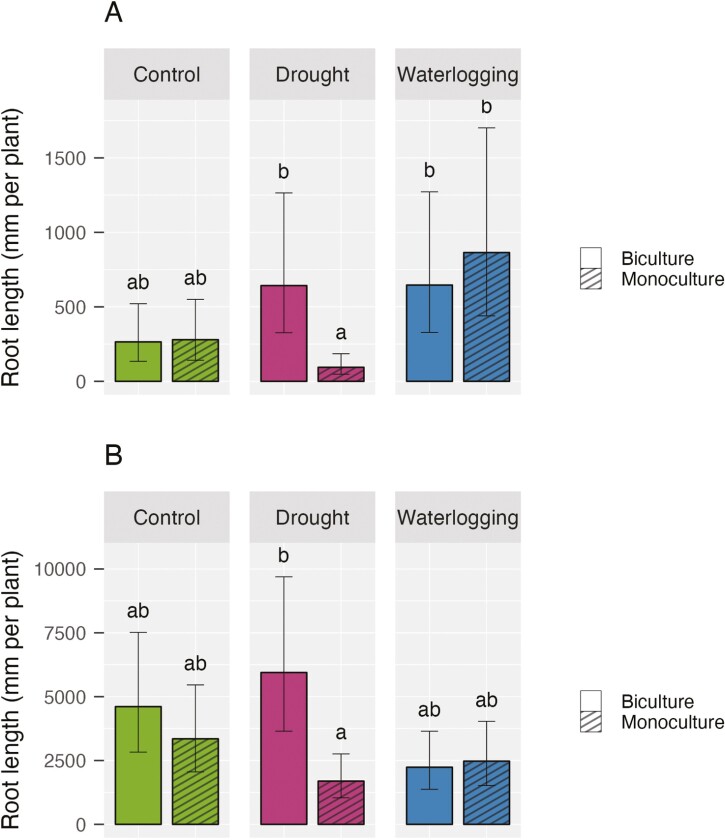
Total root length per plant (mean ± 95% confidence interval) of (A) crimson clover and (B) rye (B) under water stress by species number (*n* = 12 pots per water-species number combination).

The total root length per plant was significantly affected by an interaction between water stress and species number for both species (Table 1). Under drought, the root length per plant was 6.8 times longer when crimson clover was planted with rye compared to its monoculture counterpart (Fig. 8A). Furthermore, Tukey post-hoc tests showed that monoculture crimson clover experiencing drought had a shorter total root length per plant than the plants in both monoculture and biculture grown under waterlogging (Fig. 8A). Regarding rye, the species demonstrated a longer total root length by 3.5 times under drought when the plant was grown in a mixture compared to in monoculture (Fig. 8B).

We did not find significant main effects or interactions between water stress and species number on the main contributors to the second principal component, which were average diameter for crimson clover and maximum diameter for rye (Table 1). This suggests that the impacts of water treatments and species number on individual plants were mainly operating through root length.

## Discussion

In this greenhouse experiment, we found partial support for our hypothesis that growing cover crop species in the mixture can alleviate water stress on early plant growth, with evidence for both the sampling effect and niche complementarity. The two species’ complimentary tolerance to specific water stresses suggested the sampling effect. Meanwhile, niche complementarity became more prominent when comparing the performance of the biculture to monocultures in a water deficit condition.

Given that our study only investigated two species, we have limited power to explore the sampling effect. Even so, it is striking that our two focal species, which are among the most common cover crop species planted by farmers (CTIC 2020), exhibited opposing responses to water stress: crimson clover exhibited a tolerance to waterlogging, while rye was more resilient under drought. Drought greatly increased crimson clover’s mortality and decreased its growth rate. However, waterlogging did not affect its survival rate and biomass. Considering rye, waterlogging inhibited its growth rate, specifically after being released from stress, and reduced its per-plant biomass accumulation similar to wheat’s decreased yield under waterlogging in a three-year pot experiment by Musgrave (1994). We also observed leaf wilting in several replicates during the recovery phase, which could have resulted from waterlogging’s inhibitory effect on photosynthesis (Yang *et al.* 2020). Overall, the different tolerances of these two species to water stress provide preliminary evidence for the sampling effect. If farmers grow crimson clover and rye together, the mixture may be more likely to establish and perform stably in an uncertain climate. Future research could extend these findings to determine whether this pattern of stress tolerance applies to a wider range of grass and legume species.

We also found evidence for niche complementarity, as reflected in the performance of each species in mixture versus monoculture. Regardless of water stress, rye and crimson clover in mixture grew faster and rye accumulated more biomass per plant than in monoculture. The consistently better performance of rye in biculture corresponded to the overperformance patterns of grasses recorded by Murrell *et al.* (2017). Furthermore, under water deficit, crimson clover exhibited significantly higher biomass, and both species demonstrated longer per-plant root lengths in the mixture compared to the corresponding monocultures. These results together suggest that individual plant performance was enhanced in mixtures, particularly under drought.

In practice, ecosystem services depend on the combined performance of the plant community, and so a key question is whether sampling effects and niche complementarity result in overyielding (i.e. the average biomass of the mixture is greater than the average for monocultures) and/or transgressive yielding (i.e. the average biomass of the mixture is greater than that of the most successful species in monoculture). In field studies, best-performing cover crop mixtures usually produce similar yields to best-performing monocultures (Florence and McGuire 2020). Several studies have found evidence for overyielding but not transgressive overyielding (Smith *et al.* 2014; Finney *et al.* 2016). Interestingly, transgressive overyielding has been observed in grass–legume mixtures, but the effect was inconsistent across sites and more likely under low soil fertility (Reiss and Drinkwater 2022). Our results are largely consistent with these findings, particularly in the well-irrigated and waterlogged conditions, in which rye monoculture numerically outproduced the mixture. However, we also saw a trend under drought for the mixture to outproduce the rye monoculture, and there was no statistical difference between total biomass of the rye monoculture and the mixture under any water condition, despite the mixture containing 40% fewer rye seedlings. Given that grass–legume mixtures have evidence for transgressive overyielding in some field environments (Reiss and Drinkwater 2022) and that our study focused on the early stage of growth, it is worth investigating whether the pattern of increased performance under drought holds when extended to the full season.

The successful development of the mixture under drought could have resulted from the species’ positive interactions belowground or aboveground. Belowground, crimson clover and rye have different root types and rooting depths (Kemper *et al*. 2020). We observed in root scans that rye generally had deeper roots and longer total root length than crimson clover (Fig. 6A and B), similar to the pattern observed in a maize-faba bean intercropping system (Li *et al*. 2006). This suggests that the two species might have occupied different parts of the soil profile, reducing competition for water and allowing this limited resource to be used more effectively. The complementarity of the two species’ root architectures may also explain why they exhibited longer total root length under drought when grown in mixture vs. monoculture. Aboveground, cereals usually establish faster and grow taller than legumes. While it is still early to observe this in our experiment, in the field setting, when cereals reach the mature stage, the species creates canopy shading which can reduce water evaporation and potentially ameliorate stress for smaller plants like legumes during drought (Whitford and Duval 2020). The differences in root systems and water usage below the ground of these two focal species demonstrate resource partitioning. Moreover, rye’s potential alteration of environmental conditions aboveground suggests facilitation. We hypothesize that both mechanisms may have a positive influence on the mixture’s performance under drought. The evidence for niche complementarity in this study further suggests no competition between the two examined species in their early development. Smith *et al*. (2014) found that in their cover crop mixture, one species becomes dominant; while it contributed to a high yield of the mixture, the dominant species can potentially limit the functions of other species in the mix, which indicates possible competition among cover crop species. Indeed, interspecific competition has been observed in a similar grass–legume system: oats used as a ‘nurse crop’ for alfalfa reduce its eventual yield (Lanini *et al*. 1991). However, in this study the survival and growth of both species in the mixture was equal to or greater than their growth in monoculture.

We wish to acknowledge several limitations of this study that influence its interpretation. Turface^®^ MVP^®^ was used as the growing medium. While the substrate is not common in agricultural systems, we used it because it can be easily removed from the root systems without damaging the delicate structures, supporting root visualization (Goron *et al.* 2015). Moreover, Turface^®^ MVP^®^ can address the challenges of using field soil in containers. Field soil is slow to drain when used in pots and causes uneven distribution of water, which may introduce confounds to water stress experiments and reduce their reproducibility (Schnelle and Henderson 2017; Ogbaga *et al.* 2020). In general, greenhouse settings can have different influences on plant growth than an outdoor experiment due to several factors such as different wind conditions or growth spaces (Zhou *et al.* 2018). Moreover, a one-month duration is shorter than the actual growing period of cover crops as well as not long enough for them to fully perform their functions, for example, the crimson clover in this research did not develop its root nodules by the end of the experiment. Finally, given the wide range of species used as cover crops, results may differ depending on the species chosen and in mixes integrating more than two species. We acknowledge that the results we found for these two cover crop species may not generalize to other grass–legume mixtures. Future research can expand the scope and duration of this study to provide farmers with more concrete evidence that supports the adoption and management of cover cropping.

## Conclusion

This study found that water stress reduced the survival and growth of two cover crop species in their early development, in opposing directions: crimson clover was more tolerant to waterlogging, whereas rye demonstrated tolerance to drought. This provides evidence for the sampling effect, suggesting that given uncertain weather, farmers will be more likely to gain stable performance when growing these cover crop species together. This is especially the case because we observed no reduction in early biomass production when reducing the seeding rate of the more-productive species (rye) to accommodate the second species (crimson clover). Additionally, there was evidence for niche complementarity under drought but not waterlogging. Under drought, both species exhibited longer total root length, greater per-plant biomass and equal or greater combined biomass when they were grown in a mixture compared to monoculture. These findings suggest a potential for cover crop diversity to ameliorate drought during establishment, a possibility that deserves further study under field conditions given increasingly variable precipitation in a changing climate.

## Supporting Information

**Figure S1.** Hourly average ambient and Turface^®^ MVP^®^ temperatures throughout the course of the experiment—4 June–29 June 2021.

plae039_suppl_Supplementary_Figure_S1

## Data Availability

The data and code underlying this article are available through the Environmental Data Initiative, at https://doi.org/10.6073/pasta/6354923aa088664394193519b5d93035.
