# Supplementary material for: A mixture of grass–legume cover crop species may ameliorate water stress in a changing climate
Source: AoB Plants. 2024 Jul 25;16(4):plae039. doi: 10.1093/aobpla/plae039 (PMC11303866; doi:10.1093/aobpla/plae039)
Supplement: plae039_suppl_Supplementary_Figure_S1 [file plae039_suppl_supplementary_figure_s1.pdf]

Supporting Information

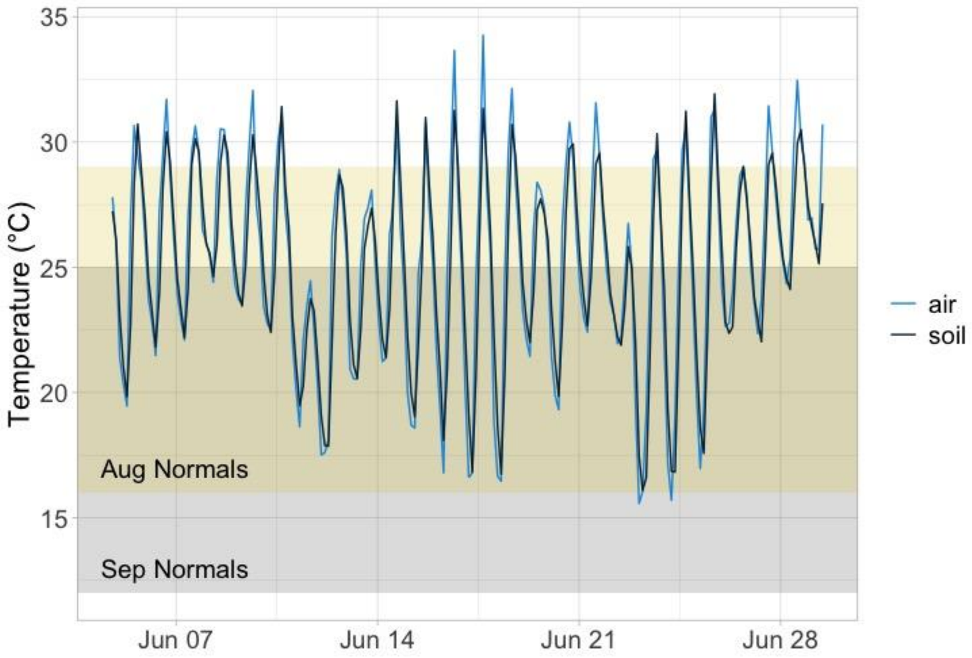

Figure S1. Hourly average ambient and Turface® MVP® temperatures throughout the course of the experiment – June 4th to June 29th, 2021. Gray and yellow areas demonstrate ranges between the maximum and minimum temperature normals in Carlisle, PA in August and September, respectively. The values were derived from SC ACIS2 using the options: first Single-Station Products, then Daily/Monthly Normals.
